# Consistent Major Differences in Sex- and Age-Specific Diagnostic Performance among Nine Faecal Immunochemical Tests Used for Colorectal Cancer Screening

**DOI:** 10.3390/cancers13143574

**Published:** 2021-07-16

**Authors:** Anton Gies, Tobias Niedermaier, Elizabeth Alwers, Thomas Hielscher, Korbinian Weigl, Thomas Heisser, Petra Schrotz-King, Michael Hoffmeister, Hermann Brenner

**Affiliations:** 1 German Cancer Research Center (DKFZ) and National Center for Tumor Diseases (NCT), Division of Preventive Oncology, 69120 Heidelberg, Germany; anton.gies@nct-heidelberg.de (A.G.); petra.schrotz-king@nct-heidelberg.de (P.S.-K.); h.brenner@dkfz-heidelberg.de (H.B.); 2 German Cancer Research Center (DKFZ), Division of Clinical Epidemiology and Aging Research, 69120 Heidelberg, Germany; elizabeth.alwers@dkfz-heidelberg.de (E.A.); korbinian-weigl@gmx.de (K.W.); t.heisser@dkfz-heidelberg.de (T.H.); m.hoffmeister@dkfz.de (M.H.); 3 German Cancer Research Center (DKFZ), Division of Biostatistics, 69120 Heidelberg, Germany; t.hielscher@dkfz-heidelberg.de; 4 German Cancer Research Center (DKFZ), German Cancer Consortium (DKTK), 69120 Heidelberg, Germany; 5 Medical Faculty Heidelberg, University of Heidelberg, 69120 Heidelberg, Germany

**Keywords:** colon cancer, faecal occult blood test, early detection, prevention

## Abstract

**Simple Summary:**

We evaluated the performance of nine faecal immunochemical tests among participants of screening colonoscopy. A total of 216 cases of advanced neoplasia (AN, colorectal cancer or advanced adenoma) and 300 randomly selected participants without AN were included. Diagnostic performance for detection of AN was assessed by sex and age (50–64 vs. 65–79 years), for each of the nine faecal immunochemical tests (FITs) individually and for all FITs combined. Major differences in diagnostic performance by sex and age were consistently seen across nine different FIT brands. Sensitivities were consistently lower, and specificities were consistently higher, for females as compared with males. Positive predictive values were similar between both sexes, but negative predictive values were higher for females. A negative FIT is less reliable in ruling out AN among men than among women and among older than among younger participants.

**Abstract:**

Evidence on diagnostic performance of faecal immunochemical tests (FITs) by sex and age is scarce. We aimed to evaluate FIT performance for detection of advanced colorectal neoplasia (AN) by sex and age across nine different FIT brands in a colonoscopy-controlled setting. The faecal samples were obtained from 2042 participants of colonoscopy screening. All eligible cases with AN (*n =* 216) and 300 randomly selected participants without AN were included. Diagnostic performance for detection of AN was assessed by sex and age (50–64 vs. 65–79 years for each of the nine FITs individually and for all FITs combined. Sensitivity was consistently lower, and specificity was consistently higher for females as compared with males (pooled values at original FIT cutoffs, 25.7% vs. 34.6%, *p* = 0.12 and 96.2% vs. 90.8%, *p* < 0.01, respectively). Positive predictive values (PPVs) were similar between both sexes, but negative predictive values (NPVs) were consistently higher for females (pooled values, 91.8% vs. 86.6%, *p* < 0.01). Sex-specific cutoffs attenuated differences in sensitivities but increased differences in predictive values. According to age, sensitivities and specificities were similar, whereas PPVs were consistently lower and NPVs were consistently higher for the younger participants. A negative FIT is less reliable in ruling out AN among men than among women and among older than among younger participants. Comparisons of measures of diagnostic performance among studies with different sex or age distributions should be interpreted with caution.

## 1. Introduction

Worldwide, colorectal cancer (CRC) accounts for approximately 1 million new cases among men and for approximately 800,000 new cases among women annually [1]. Faecal immunochemical tests (FITs) are widely recommended [2,3] and used [4,5] for population-wide screening and early detection of CRC and its precancerous lesions. The diagnostic performance of quantitative FITs has been assessed in many studies and has been summarized in meta-analyses [6,7]; however, evidence on FIT performance according to sex and age derived from colonoscopy-controlled studies is scarce and limited to only a few FIT brands [8]. Furthermore, it is unclear whether differences exist for detection of advanced colorectal neoplasia (AN) by sex or by age across different FIT brands.

We aimed to evaluate the diagnostic performance of a large number of different quantitative FITs according to sex and to age, using faecal samples obtained from individuals undergoing colonoscopy screening in Germany.

## 2. Materials and Methods

The Standards for the Reporting of Diagnostic Accuracy Studies (STARD) [9] and the standard for Faecal Immunochemical Tests for Haemoglobin Evaluation Reporting (FITTER) [10] were followed.

### 2.1. Study Design and Population

This analysis was carried out following a direct comparison and combination of nine quantitative FITs for detection of AN, details of which have been published previously [11,12]. Briefly, this study is based on the BLITZ study, which has been running since 2005 with the aim to collect blood and stool samples among average-risk individuals before undergoing colonoscopy screening for evaluation of novel non-invasive CRC screening tests. Study participants are informed and recruited during their preparatory visit (typically 1 week before colonoscopy) in cooperating gastroenterology practices in southwest Germany.

The study was approved by the Ethics committee of University of Heidelberg (178/2005) and those of the state chambers of physicians of Baden-Wuerttemberg (M118-05-f), Rhineland-Palatinate (837.047.06(5145)) and Saarland (217/13). The BliTz study was registered in the German Clinical Trials Register (DRKSID: DRKS00008737). Written informed consent was obtained from each study participant. Further information about the BLITZ study has been provided elsewhere [13,14,15].

### 2.2. Selection of Study Participants

A total of 2042 participants, who were recruited until 2010 and who provided faecal samples in stool containers (60 mL), were eligible for this project. After excluding participants <50 or ≥80 years of age (*n =* 52), with inflammatory bowel disease (*n =* 10), history of previous colorectal neoplasia (*n =* 39), colonoscopy in the past 5 years (*n =* 114), stool sample collection not prior to colonoscopy (*n =* 75), and incomplete colonoscopy (*n =* 8) or inadequate bowel cleansing (*n =* 77), 1667 participants fulfilled the inclusion criteria for this analysis. All participants diagnosed with AN, i.e., either CRC or advanced adenoma (AA, defined as adenomas with either size ≥1 cm, villous/tubulovillous components, or high-grade dysplasia) who provided enough faeces for the evaluation of 9 FITs were included (*n =* 216). For one FIT (immoCARE-C), the analyses were based on one less AN case (*n =* 215), because one FIT measurement was missing. To save resources and capacities, 300 participants without CRC and AA were randomly selected and included for specificity calculations.

### 2.3. Data/Sample Collection and Processing

Participants were asked to collect a faecal sample before starting bowel preparation for colonoscopy, to store the sample in a freezer (or, if not possible, in a refrigerator), and to bring it in a temperature-isolated bag to the gastroenterology practice on the day of the scheduled colonoscopy. In the practices, the samples were kept at −20 °C and sent on dry ice to a central laboratory, and afterwards to the German Cancer Research Center (DKFZ, Heidelberg) for final storage at −80 °C. Although this preanalytical sample procedure differs from the recommended faecal sampling procedure (i.e., filling the faecal sampling tubes directly with fresh stool), we have found in a recent retrospective analysis that estimates of diagnostic performance of FITs remained fairly stable even after long-term frozen storage and repeat thawing and freezing cycles [16].

The screening colonoscopy was performed by experienced colonoscopists who were unaware of the FIT result. Afterwards, colonoscopy (and histology) reports were collected, and relevant data were extracted by trained medical data officers who were likewise blinded to any FIT result.

### 2.4. Test Analysis

Faecal samples were thawed in 2016 in order to measure different FITs in parallel, as previously described [11,12]. Overall, 516 faecal samples from average-risk participants of screening colonoscopy were measured using nine quantitative FITs from seven manufactures. All FITs were approved for use in Germany. Detailed test characteristics are shown in Table S1. Before filling the single faecal sample collection tubes, the stool within each container was mixed to reduce heterogeneity in faecal haemoglobin distribution [17]. All nine FITs were evaluated simultaneously under the same preanalytical and analytical conditions: Stool specimens were extracted for the nine FITs using the special sampling tubes that had been designed to transfer a defined amount of faeces into a haemoglobin-stabilizing buffer of the tube. Afterwards, the tubes were shaken and kept at ambient temperature (range 20–24 °C) until they were blindly measured on the next day. Further detailed information on test analysis has been published previously [11,12].

### 2.5. Statistical Analysis

All quantitative faecal haemoglobin measurements were converted to the same, and directly comparable, unit of µg haemoglobin per gram faeces (µg/g) [18].

Sensitivities, specificities, positive and negative predictive values (PPVs and NPVs) with their 95% confidence interval (CI) were calculated for detection of AN (either CRC or AA) by sex and by age (50–64 and 65–79 years). The analyses were conducted at thresholds recommended by the manufacturers (=original thresholds, range 2–17 µg/g) and at thresholds yielding an equal overall specificity of 95%. In addition, positivity rates with their 95% CIs were computed. Due to the overrepresentation of participants with AN (all AN cases, *n* = 216) in comparison to the participants without AN (random sample, *n* = 300) by design, PPVs, NPVs and positivity rates were derived from weighted analyses. Weights were calculated by dividing original fractions, which were observed among the 1667 eligible study participants, by sampling fractions for inclusion in the FIT. This way, positivity rates, PPVs, and NPVs reflect the prevalence of AN, sex, and age distribution observed in the cohort of eligible participants (*n =* 1667) who fulfilled the inclusion criteria. Testing for statistical differences by sex and by age was conducted using logistic regression models. For positivity rates, PPVs, and NPVs, a weighted logistic regression model was fitted. *p*-values and CIs were based on the Wald test.

Generalized estimating equations (GEE) logistic regression models were used to derive pooled estimates including 95% CIs of the various measures of diagnostic performance by sex and by age across the nine FITs and to test for the associations of age and sex with diagnostic performance, taking FIT effects and dependency of observations within the same individuals into account. Statistical testing by sex and by age was conducted using the Wald test.

To assess the overall diagnostic performance within the clinically relevant segment of ≥80% specificity, partial areas under the curves (AUCs) were calculated in such a way that they became 50% for nondiscriminant and 100% for perfectly discriminating tests. Derivation of 95% CIs and testing for statistical significance of differences in partial AUCs were done using 2000 bootstrap replicates.

Two-sided *p*-values that were below 0.05 were considered statistically significant. The analyses of partial AUCs were conducted using R (version 3.6.0, R Core Team, Vienna, Austria) with the R package ‘pROC’ (version 1.16.2), whereas all other analyses and statistical tests were conducted using SAS enterprise guide (version 7.1, Cary, NC, USA).

## 3. Results

### 3.1. Study Population

The characteristics of all eligible 1667 participants of colonoscopy screening are shown in Table 1. The sample included approximately equal numbers of women and men. The age distribution was similar among both sexes, and most participants were between 50 and 64 years old. Advanced neoplasia was detected in 230 participants. Among these, colorectal cancer and advanced adenomas were the most advanced finding of colonoscopy screenings for 16 (1.0%) and 214 (12.8%) participants. The overall AN prevalence was 13.8% in the total study population. It was higher among men (17.3%) than among women (10.0%) and in the age group 65–79 (17.6%) than in the age group 50–64 (11.3%).

### 3.2. Diagnostic Performance by Sex

**Figure 1:**
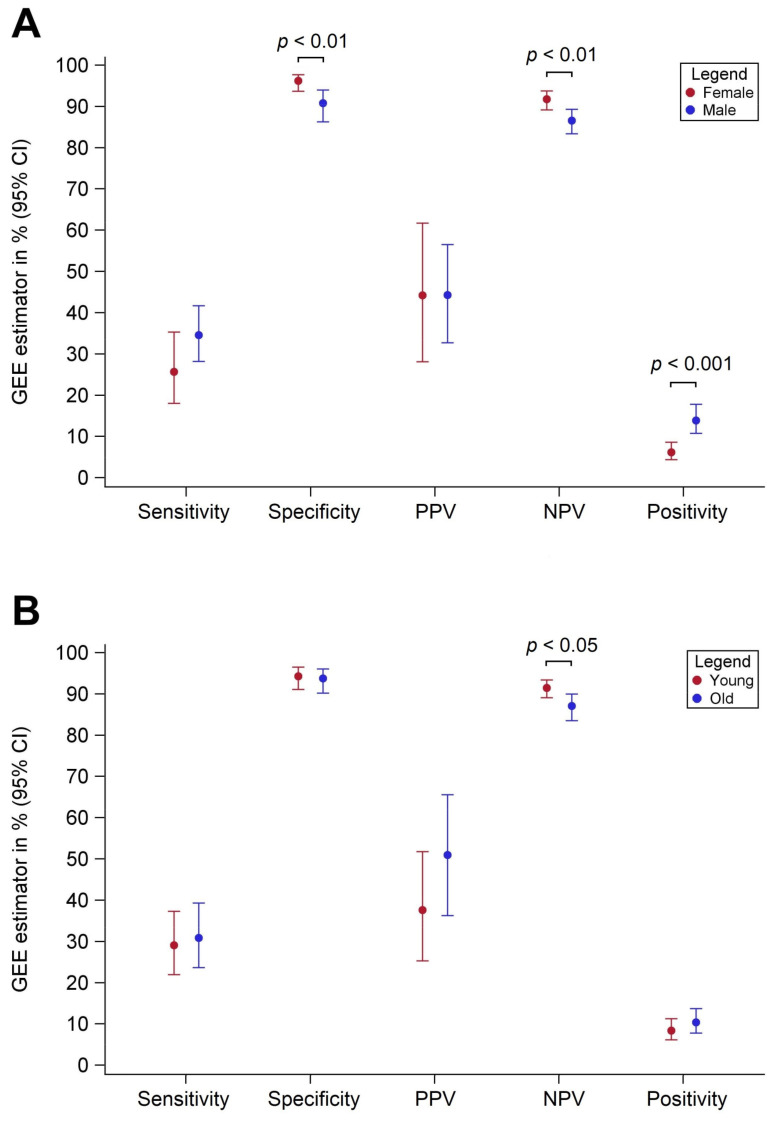
Diagnostic performance parameters by sex (**panel A**) and age (**panel B**) for detection of advanced neoplasia pooled across 9 FIT brands at original positivity thresholds.

Across all FITs and all assessed thresholds, sensitivities were consistently lower and specificities were consistently higher among females as compared with among males (Table 2). At original threshold values, substantial differences in measures of diagnostic performance were observed among the different single FIT brands. However, when threshold values were adjusted to yield identical levels of overall specificity (to enhance the comparability between the FITs), no meaningful differences were observed among the different FIT brands. At original thresholds, pooled sensitivities were 25.7% vs. 34.6% (*p* = 0.12) and pooled specificities were 96.2% vs. 90.8% (*p* = 0.005) for females and males, respectively. Similar sex differences were observed at thresholds adjusted to yield equal overall specificities (95%) across the FITs. PPVs were similar between both sexes, but NPVs were consistently higher for females (pooled values 91.8% vs. 86.6%, *p* < 0.01) (Table 3). Differences in sensitivities diminished when using sex-specific cutoffs, whereas differences in PPVs increased. Pooled sex-specific differences in sensitivity, specificity, PPV, NPV, and positivity at original thresholds are summarized in Figure 1A.

The overall diagnostic performance, measured by the partial AUC (between 80% and 100% specificity), showed no clinically relevant difference between men and women (Table S2). For four of the nine FITs, partial AUCs were slightly higher for men (up to 3.1%), whereas for the other five FITs, the partial AUCs were slightly higher for women (up to 2.4%), but none of these small differences reached statistical significance.

### 3.3. Diagnostic Performance by Age

Pooled age-specific differences in sensitivity, specificity, PPV, NPV, and positivity are summarized in Figure 1B. Differences in sensitivity and specificity between younger (50–64 years) and older (65–79 years) study participants were generally small (Table 4) and less consistent than differences according to sex. Similar results were observed at thresholds adjusted to yield an overall specificity of 95%, and none of these differences between both age groups was statistically significant.

PPVs were consistently higher among the older age group as compared with the younger age group (Table 5), but differences were not statistically significant. At original thresholds, pooled PPVs were 37.6% and 51.0% (*p* = 0.16) for the younger and older age groups, respectively. NPVs were consistently lower (by about 5%) for the older age group, and pooled estimates were statistically significantly different (*p* = 0.02) between both age groups, across all thresholds. Differences in NPVs were slightly larger when age-specific cutoffs were used (about 6%).

Partial AUCs were slightly higher (up to 2.7%) for the younger study group for five of the nine FITs, and for the other four FITs these estimates were slightly higher (up to 3.8%) for the older study group, but none of these differences was statistically significant (Table S2).

## 4. Discussion

In this study, we assessed the diagnostic performance for detection of AN of nine different quantitative FITs according to sex and age, using stool samples collected from average-risk participants of screening colonoscopy. Even when adjusting FIT cutoffs to yield equal specificity in the entire study population, pooled sensitivities were consistently higher, whereas pooled specificities were statistically significantly lower among males as compared with females. Pooled PPVs were very similar between both sexes. By contrast, pooled NPVs were statistically significantly lower for males, suggesting that a negative FIT is less reliable in ruling out AN among men than among women. When using sex-specific cutoffs with respect to specificities, differences in sensitivities by sex diminished, whereas differences in PPVs and NPVs became greater. According to age, pooled sensitivities and specificities were very similar between both age groups, but pooled PPVs were consistently higher and pooled NPVs were statistically significantly lower for the older age group.

To the best of our knowledge, this is the first study to assess diagnostic performance of several different FIT brands in parallel for detection of AN by sex among participants of colonoscopy screening. There are only a few previous studies that have investigated FIT performance for AN detection with respect to sex [19,20,21,22]. Each of these studies assessed only one specific FIT brand and consistently reported higher sensitivity for men than for women, although the magnitude of the difference varied among studies. Specificities were generally lower among men, but sex differences were generally smaller for specificity, varying only by a few percent units. It was unclear, however, to what extent differences in the magnitude of sex- and age-specific variations were due to differences in study populations and age groups or to differences in FIT brands assessed in these studies. Our study demonstrates across several different FIT brands consistently higher sensitivities (by 3–13% units) along with consistently lower specificities (by 2–10% units) for men as compared with women at original cutoffs. Differences persisted when adjusting to equal specificity in the entire study population, but diminished when using equal specificities among women and among men, respectively.

It remains to be investigated by future studies if age and sex are similarly, or possibly more strongly, associated with FIT performance among symptomatic patients than among screening participants. Symptomatic patients may comprise a very heterogeneous group and it is conceivable that differences in performance characteristics vary in strength or even direction across heterogeneous symptomatic groups (e.g., those reporting abdominal pain vs. change in bowel habits). In both symptomatic and screening populations, it should be considered that age and sex may interact or be associated with other covariates potentially influencing FIT results. For example, intake of proton pump inhibitors (PPIs) has been suggested to be associated with reduced accuracy of FIT by some [23,24] but not all [25] studies. Furthermore, interactions with sex or intake of other drugs have been suggested [26].

The reasons for the higher sensitivity and lower specificity of FITs among men than among women at equal cutoffs remain to be fully explored. Possible reasons may include the higher proportion of AN located in the distal colon and rectum that are more frequently detected by FIT than proximal AN [27,28], higher rates of aspirin use for cardio prevention [29], and a shorter colonic transit time [30] that may be associated with less Hb degradation prior to defecation. The higher positivity rate and the lower NPV among men might also be partly explained by the higher prevalence of AN among men than among women (17.3% versus 10.0% in our study population).

We are aware of only three previous studies that assessed the FIT performance for AN detection among participants of colonoscopy screening according to age [20,22,31]. Again, each of these studies assessed only one specific FIT brand. Furthermore, they were conducted in very different study populations, used different age categorizations and yielded inconsistent results. In our study, no consistent differences in sensitivity and specificity were found across nine different FIT brands evaluated in parallel in the same study population. However, PPVs consistently tended to be higher and NPVs consistently tended to be lower among older as compared with younger age groups. Given the lack of differences in sensitivity and specificity, the differences in PPVs and NPVs most likely are due to differences in prevalence of advanced adenomas, which is higher in older than in younger participants of colonoscopy screening (17.6% versus 11.3% in our study population).

Finally, but importantly, eight out of nine FITs yielded very similar overall measures of diagnostic performance, as quantified by partial AUCs. Slightly lower partial AUCs were observed only for QuikRead go iFOBT, but this observation was caused by the lower analytical working limit being unusually high (15 µg/g) for this FIT. Furthermore, partial AUCs were very similar between sexes and age groups for each of the nine FIT brands. Therefore, no clinically relevant differences in overall diagnostic performance by sex and age were observed between FITs from different manufacturers.

A major strength of our study is the parallel measurement of the faecal haemoglobin concentration across nine different quantitative FITs under the same preanalytical and analytical test requirements in a colonoscopy-controlled study setting. The few previous studies that assessed the diagnostic performance according to sex [19,20,21,22] and age [20,22] included only a single FIT brand each. A further strength is that stool samples were collected from participants of colonoscopy screening prior to bowel preparation and the samples were stored in the same manner until parallel laboratory test analysis. Furthermore, the results of colonoscopy screening with adequate bowel preparation served as a reference standard to calculate diagnostic performance. In order to enhance comparability of diagnostic performance by sex and age across different FITs, we adjusted the cutoffs to yield equal overall specificities.

Our study also has limitations. Although more than 2000 participants of colonoscopy screening were recruited, the limited overall number of AN cases (*n =* 216) and randomly selected controls (*n =* 300) did not allow for in-depth analyses for each sex- and age-specific subset, for example, stratified by adenoma location. Despite the limited numbers, the pooled results of the GEE model revealed statistically significant differences in overall specificity, NPV, and positivity rate. The suggested differences in sensitivities warrant further research with larger case numbers. Future studies should also investigate potential mechanisms by which FIT sensitivity varies across groups of participants, for example, prevalence of anemia according to age and sex.

## 5. Conclusions

In conclusion, we observed consistently higher sensitivities and lower specificities among males as compared with among females with a number of different FITs and a broad range of threshold values. Furthermore, the analyses among men yielded consistently higher positivity rates, comparable PPVs, and lower NPVs than among women. According to age, no major differences in sensitivity and specificity were observed, but positive and negative predictive values differed, probably reflecting differences in AN prevalence between sexes and age groups. A negative FIT is less reliable for ruling out AN among men than among women and among older than among younger individuals. Further studies should address if, and to what extent, the sex- and age-specific differences might be relevant for the design of screening offers and interpretation of FIT results in various groups of screening participants, for example, by using sex- and age-specific cutoffs. These questions could, for example, be addressed in comprehensive modelling studies for which our results provide important input information. The diagnostic performance of FITs should be interpreted and compared with caution among studies with different sex or age distributions.

## Figures and Tables

**Table 1:** Characteristics of the study population.

Characteristic		*n*	Col %	FIT Measurements	
				*n*	Row %
Sex	Women	806	48.4		
	Men	861	51.6		
Age	50–64	1014	60.8		
	65–79	653	39.2		
Advanced Neoplasia	Yes	230	13.8	216 *	93.9 *
	Colorectal cancer	16	1.0	16 *	
	Advanced adenoma	214	12.8	200 *	
	No	1437	86.2	300 **	20.9 **

* All participants with sufficient stool for conducting 9 FITs. ** Random sample.

**Table 2:** Sensitivity and specificity for detection of advanced neoplasia by sex.

FIT Brand	Sensitivity (%)			Specificity (%)				
	Female	Male	Diff.	*p*	Female	Male	Diff.	*p*
At original thresholds recommended by the manufacturers								
IDK Hb ELISA	40.5	49.3	−8.8	0.22	90.3	80.7	+9.6	**0.02**
QuantOn Hem	36.5	48.6	−12.1	0.09	89.0	82.1	+6.9	0.09
immoCARE-C	33.8	41.1	−7.3	0.29	92.9	86.9	+6.0	0.09
CAREprime	28.4	38.0	−9.6	0.16	95.5	86.9	+8.6	**0.01**
RIDASCREEN Hb	32.4	43.0	−10.6	0.13	94.8	86.2	+8.6	**0.01**
Eurolyser FOB test	17.6	25.4	−7.8	0.20	98.7	95.2	+3.5	0.09
OC-Sensor	17.6	23.9	−6.3	0.28	99.4	95.9	+3.5	0.08
QuikRead go iFOBT	14.9	25.4	−10.5	0.08	98.7	94.5	+4.2	0.06
SENTiFIT-FOB Gold	17.6	23.9	−6.3	0.28	98.7	93.8	+4.9	**0.04**
**GEE-Model**	25.7	34.6	−8.9	0.12	96.2	90.8	+5.4	**0.005**
At adjusted thresholds yielding 95% specificity among all study participants								
IDK Hb ELISA	25.7	33.8	−8.1	0.22	96.8	93.1	+3.7	0.15
QuantOn Hem	24.3	27.5	−3.2	0.62	95.5	94.5	+1.0	0.69
immoCARE-C	27.0	34.0	−7.0	0.29	96.8	93.1	+3.7	0.15
CAREprime	18.9	28.2	−9.3	0.14	98.1	91.7	+6.4	**0.02**
RIDASCREEN Hb	25.7	36.6	−10.9	0.11	96.1	93.8	+2.3	0.36
Eurolyser FOB test	21.6	28.2	−6.6	0.30	98.1	91.7	+6.4	**0.02**
OC-Sensor	20.3	28.9	−8.6	0.17	97.4	92.4	+5.0	0.06
QuikRead go iFOBT	14.9	25.4	−10.5	0.08	98.7	94.5	+4.2	0.06
SENTiFIT-FOB Gold	23.0	28.9	−5.9	0.35	98.1	91.7	+6.4	**0.02**
**GEE-Model**	22.4	30.2	−7.8	0.18	97.3	93.0	+4.3	**0.04**
At adjusted thresholds yielding 95% specificity among women and among men, respectively								
IDK Hb ELISA	28.4	24.6	3.8	0.55	94.8	95.2	−0.4	0.89
QuantOn Hem	29.7	25.4	4.3	0.49	94.8	95.2	−0.4	0.89
immoCARE-C	32.4	23.4	9.0	0.16	94.8	95.2	−0.4	0.89
CAREprime	31.1	23.9	7.2	0.26	94.8	95.2	−0.4	0.89
RIDASCREEN Hb	32.4	24.6	7.8	0.22	94.8	95.2	−0.4	0.89
Eurolyser FOB test	25.7	26.1	−0.4	0.95	96.1	95.2	+0.9	0.68
OC-Sensor	27.0	26.1	0.9	0.88	94.8	95.2	−0.4	0.89
QuikRead go iFOBT	14.9	25.4	−10.5	0.08	98.7	95.1	+3.6	0.09
SENTiFIT-FOB Gold	24.3	23.9	0.4	0.95	96.8	95.2	+1.6	0.48
**GEE-Model**	27.3	24.8	2.5	0.66	95.6	95.2	+0.4	0.82

Abbreviations: CI, confidence interval; FIT, faecal immunochemical test; GEE, generalized estimating equations; Hb, haemoglobin. Bold numerals: statistically significant differences (*p* < 0.05).

**Table 3:** Positive (PPV) and negative (NPV) predictive values for detection of advanced neoplasia by sex.

FIT Brand	PPV (% (95% CI))				NPV (% (95% CI))			
	Female	Male	Diff.	*p*	Female	Male	Diff.	*p*
At original thresholds recommended by the manufacturers								
IDK Hb ELISA	31.9	35.1	−3.2	0.75	92.8	88.0	4.8	0.10
QuantOn Hem	27.4	36.7	−9.3	0.37	92.3	88.1	4.2	0.16
immoCARE-C	35.5	39.7	−4.2	0.73	92.3	87.3	5.0	0.09
CAREprime	41.6	38.6	3.0	0.83	92.0	86.7	5.3	0.08
RIDASCREEN Hb	41.1	40.1	1.0	0.94	92.3	87.5	4.8	0.10
Eurolyser FOB test	63.9	56.9	7.0	0.75	91.1	85.6	5.5	0.06
OC-Sensor	76.6	57.1	19.5	0.41	91.2	85.5	5.7	0.06
QuikRead go iFOBT	57.9	51.3	6.6	0.77	90.9	85.5	5.4	0.08
SENTiFIT-FOB Gold	63.1	47.4	15.7	0.47	91.1	85.2	5.9	**0.05**
**GEE-Model**	44.2	44.3	−0.1	1.00	91.8	86.6	5.2	**0.01**
At adjusted thresholds yielding 95% specificity among all study participants								
IDK Hb ELISA	46.8	50.9	−4.1	0.80	92.1	87.0	5.1	0.08
QuantOn Hem	37.1	51.8	−14.7	0.36	91.8	86.2	5.6	**0.05**
immoCARE-C	48.2	50.4	−2.2	0.89	92.2	87.1	5.1	0.07
CAREprime	51.8	40.7	11.1	0.56	91.5	85.9	5.6	**0.05**
RIDASCREEN Hb	42.3	55.2	−12.9	0.41	92.0	87.6	4.4	0.12
Eurolyser FOB test	55.3	41.1	14.2	0.44	91.8	85.9	5.9	**0.04**
OC-Sensor	46.4	44.3	2.1	0.91	91.6	86.1	5.5	0.06
QuikRead go iFOBT	55.8	48.1	7.7	0.73	91.2	85.8	5.4	0.06
SENTiFIT-FOB Gold	56.9	41.3	15.6	0.39	91.9	86.0	5.9	**0.04**
**GEE-Model**	47.6	46.9	0.7	0.96	91.8	86.4	5.4	**0.005**
At adjusted thresholds yielding 95% specificity among women and among men, respectively								
IDK Hb ELISA	37.8	50.9	−13.1	0.41	92.2	85.7	6.5	**0.03**
QuantOn Hem	38.8	52.8	−14.0	0.37	92.3	85.9	6.4	**0.03**
immoCARE-C	41.0	49.4	−8.4	0.59	92.6	85.6	7.0	**0.02**
CAREprime	41.0	50.2	−9.2	0.52	92.5	85.6	6.9	**0.02**
RIDASCREEN Hb	41.1	50.9	−9.8	0.53	92.6	85.7	6.9	**0.02**
Eurolyser FOB test	42.4	51.7	−8.3	0.58	92.0	86.0	6.0	**0.04**
OC-Sensor	36.6	51.7	−15.1	0.34	92.1	86.0	6.1	**0.04**
QuikRead go iFOBT	55.8	51.6	4.2	0.85	91.2	85.9	5.3	0.07
SENTiFIT-FOB Gold	45.6	49.0	−3.4	0.84	91.9	85.6	6.3	**0.03**
**GEE-Model**	40.9	50.9	−10.0	0.44	92.1	85.8	6.3	**0.001**

Abbreviations: CI, confidence interval; FIT, faecal immunochemical test; GEE, generalized estimating equations; Hb, haemoglobin. Bold numerals: statistically significant differences (*p* < 0.05).

**Table 4:** Sensitivity and specificity for detection of advanced neoplasia by age.

FIT Brand	Sensitivity (% (95% CI))				Specificity (% (95% CI))			
	50–64 Years	65–79 Years	Diff.	*p*	50–64 Years	65–79 Years	Diff.	*p*
At original thresholds recommended by the manufacturers								
IDK Hb ELISA	44.0	48.6	−4.6	0.50	88.4	81.5	6.9	0.10
QuantOn Hem	43.1	45.8	−2.7	0.69	86.2	84.9	1.3	0.75
immoCARE-C	37.0	40.2	−3.2	0.64	90.6	89.1	1.5	0.67
CAREprime	32.1	37.4	−5.3	0.42	91.2	91.6	−0.4	0.90
RIDASCREEN Hb	35.8	43.0	−7.2	0.28	93.4	86.6	6.8	**0.05**
Eurolyser FOB test	21.1	24.3	−3.2	0.58	96.1	98.3	−2.2	0.29
OC-Sensor	21.1	22.4	−1.3	0.81	97.2	98.3	−1.1	0.55
QuikRead go iFOBT	22.0	21.5	0.5	0.93	96.1	97.5	−1.4	0.53
SENTiFIT-FOB Gold	22.9	20.6	2.3	0.67	95.6	97.5	−1.9	0.40
**GEE-Model**	29.1	30.9	−1.8	0.73	94.3	93.8	0.5	0.76
At adjusted thresholds yielding 95% specificity among all study participants								
IDK Hb ELISA	28.4	33.6	−5.2	0.41	95.6	94.1	1.5	0.57
QuantOn Hem	24.8	28.0	−3.2	0.59	95.6	94.1	1.5	0.57
immoCARE-C	28.7	34.6	−5.9	0.35	95.6	94.1	1.5	0.57
CAREprime	22.9	27.1	−4.2	0.48	94.5	95.8	−1.3	0.61
RIDASCREEN Hb	30.3	35.5	−5.2	0.41	95.6	94.1	1.5	0.57
Eurolyser FOB test	23.9	28.0	−4.1	0.48	95.0	95.0	0.0	0.98
OC-Sensor	25.7	26.2	−0.5	0.94	96.1	93.3	2.8	0.27
QuikRead go iFOBT	22.0	21.5	0.5	0.93	96.1	97.5	−1.4	0.53
SENTiFIT-FOB Gold	23.9	29.9	−6.0	0.32	94.5	95.8	−1.3	0.61
**GEE-Model**	25.6	29.4	−3.8	0.49	95.4	94.9	0.5	0.80
At adjusted thresholds yielding 95% specificity among younger and older participants, respectively								
IDK Hb ELISA	30.3	21.5	8.8	0.14	95.0	95.0	0.0	0.98
QuantOn Hem	25.7	27.1	−1.4	0.81	95.0	95.0	0.0	0.98
immoCARE-C	29.6	28.0	1.6	0.80	95.0	95.0	0.0	0.98
CAREprime	22.9	28.0	−5.1	0.39	95.0	95.0	0.0	0.98
RIDASCREEN Hb	31.2	22.4	8.8	0.15	95.0	95.0	0.0	0.98
Eurolyser FOB test	23.9	33.6	9.7	0.11	95.0	95.0	0.0	0.98
OC-Sensor	27.5	23.4	4.1	0.48	95.0	95.0	0.0	0.98
QuikRead go iFOBT	22.0	21.5	0.5	0.93	96.1	97.5	−1.4	0.53
SENTiFIT-FOB Gold	23.9	34.6	−10.7	0.08	95.0	95.0	0.0	0.98
**GEE-Model**	26.3	26.7	−0.4	0.95	95.2	95.2	0.0	0.97

Abbreviations: CI, confidence interval; FIT, faecal immunochemical test; GEE, generalized estimating equations; Hb, haemoglobin. Bold numerals: statistically significant differences (*p* < 0.05).

**Table 5:** Positive (PPV) and negative (NPV) predictive value for detection of advanced neoplasia by age.

FIT Brand	PPV (% (95% CI))				NPV (% (95% CI))			
	50–64 Years	65–79 Years	Diff.	*p*	50–64 Years	65–79 Years	Diff.	*p*
At original thresholds recommended by the manufacturers								
IDK Hb ELISA	31.7	35.4	−3.7	0.70	92.6	88.4	4.2	0.15
QuantOn Hem	26.7	37.5	−10.8	0.26	92.2	88.2	4.0	0.17
immoCARE-C	32.3	43.1	−10.8	0.35	92.0	87.8	4.2	0.14
CAREprime	31.5	49.4	−17.9	0.15	91.4	87.6	3.8	0.20
RIDASCREEN Hb	40.3	40.9	−0.6	0.96	92.0	87.9	4.1	0.15
Eurolyser FOB test	41.8	76.5	−34.7	0.07	90.6	86.3	4.3	0.14
OC-Sensor	54.7	78.3	−23.6	0.21	90.8	86.1	4.7	0.11
QuikRead go iFOBT	42.8	65.9	−23.1	0.22	90.7	85.7	5.0	0.09
SENTiFIT-FOB Gold	42.9	67.2	−24.3	0.19	90.8	85.7	5.1	0.08
**GEE-Model**	37.6	51.0	−13.4	0.16	91.5	87.1	4.4	**0.02**
At adjusted thresholds yielding 95% specificity among all study participants								
IDK Hb ELISA	44.4	55.0	−10.6	0.48	91.2	86.9	4.3	0.13
QuantOn Hem	41.8	50.4	−8.6	0.58	90.8	85.9	4.9	0.10
immoCARE-C	44.1	55.5	−11.4	0.45	91.3	87.0	4.3	0.14
CAREprime	33.3	57.7	−24.4	0.13	90.5	85.9	4.6	0.13
RIDASCREEN Hb	46.0	56.1	−10.1	0.50	91.4	87.2	4.2	0.14
Eurolyser FOB test	36.6	54.2	−17.6	0.26	90.7	86.0	4.7	0.12
OC-Sensor	44.6	45.2	−0.6	0.97	91.0	85.5	5.5	0.07
QuikRead go iFOBT	40.8	64.3	−23.5	0.21	90.6	85.3	5.3	0.08
SENTiFIT-FOB Gold	34.2	60.2	−26	0.10	90.6	86.4	4.2	0.16
**GEE-Model**	40.5	54.9	−14.4	0.25	90.9	86.2	4.7	**0.02**
At adjusted thresholds yielding 95% specificity among younger and older participants, respectively								
IDK Hb ELISA	43.0	54.3	−11.3	0.26	91.4	84.9	6.5	**0.03**
QuantOn Hem	39.6	53.3	−13.7	0.38	90.9	85.9	5.0	0.09
immoCARE-C	41.9	53.9	−12.0	0.44	91.4	86.0	5.4	0.07
CAREprime	35.7	54.1	−18.4	0.24	90.6	86.0	4.6	0.13
RIDASCREEN Hb	43.7	48.6	−4.9	0.76	91.5	85.1	6.4	**0.03**
Eurolyser FOB test	36.6	58.7	−22.1	0.15	90.7	87.0	3.7	0.21
OC-Sensor	40.7	49.5	−8.8	0.58	91.1	85..2	5.9	0.05
QuikRead go iFOBT	40.8	64.3	−23.5	0.21	90.6	85.3	5.3	0.08
SENTiFIT-FOB Gold	36.6	59.4	−22.8	0.14	90.7	87.1	3.6	0.23
**GEE-Model**	39.9	54.3	−14.4	0.26	91.0	85.8	5.2	**0.01**

Abbreviations: CI, confidence interval; FIT, faecal immunochemical test; GEE, generalized estimating equations; Hb, haemoglobin. Bold numerals: statistically significant differences (*p* < 0.05).

## Data Availability

The data supporting reported results can be made available upon reasonable request.

## Supplementary Materials

The following are available online at https://www.mdpi.com/article/10.3390/cancers13143574/s1, Table S1: Test characteristics, Table S2: Partial area under the curve (% (95% CI)) for detection of advanced neoplasia by sex and by age.
