# Supplementary material for: Consistent Major Differences in Sex- and Age-Specific Diagnostic Performance among Nine Faecal Immunochemical Tests Used for Colorectal Cancer Screening
Source: Cancers (Basel). 2021 Jul 16;13(14):3574. doi: 10.3390/cancers13143574 (PMC8306133; doi:10.3390/cancers13143574)
Supplement: Supplementary file 1 [file cancers-13-03574-s001.zip › cancers-1235703-supplementary.pdf]

Supplementary Materials: Consistent Major Differences in Sex- and Age-Specific Diagnostic Performance among Nine Faecal Immunochemical Tests Used for Colorectal Cancer Screening

Anton Gies, Tobias Niedermaier, Elizabeth Alwers, Thomas Hielscher, Korbinian Weigl, Thomas Heisser, Petra Schrotz-King, Michael Hoffmeister and Hermann Brenner

Table S1. Test characteristics.

FIT brand	Manufacturer (City, Country)	Stool sample collection tube (fecal mass/buffer volume)	Analyzer	Quantitative analytical range [µg Hb/g feces]	Positivity threshold [µg Hb/g feces]		
					Original	At 96.7% specificity	At 93.0% specificity*
IDK Hb ELISA	Immundiagnostik (Bensheim, Germany)	IDK Extract (15 mg/1.5 mL)	ELISA reader	0.09 to 50.0	2.0	15.32	4.8
QuantOn Hem	Immundiagnostik (Bensheim, Germany)	QuantOn Hem TUBE (15 mg/1.5 mL)	QuantOn Hem test cassette + Smartphone	0.3 to 100.0	3.7	17.73	9.59
immoCARE-C	CARE diagnostica (Möllersdorf, Austria)	Sample Collection Tube (20 mg/2.5 mL)	immoCARE-C test cassette + CAREcube	3.75 to 250.0	6.25	17.3	9.2
CAREprime	Alfresa Pharma (Osaka, Japan)	Specimen Collection Container A (9.5 mg/1.9 mL)	CAREprime	1.6 to 240.0	6.3	12.35	6.65
RIDASCREEN Hb	R-Biopharm (Darmstadt,Germany)	RIDA TUBE Haemoglobin (10 mg/2.5 mL)	ELISA reader	0.65 to 50.0	8.0	29.54	12.27
Eurolyser FOB test	Eurolyser Diagnostica	Eurolyser FOB Sample Collector (19.9 mg/1.6 mL)	Eurolyser CUBE	2.01 to 80.4	8.04	6.11	2.01

	(Salzburg, Austria)						
OC-Sensor	Eiken Chemical (Tokyo, Japan)	OC Auto-Sampling Bottle 3 (10 mg/2.0 mL)	OC-Sensor io	10.0 to 200.0	10.0	6.6	3.6
QuikRead go iFOBT	Orion Diagnostica (Espoo, Finland)	QuikRead go iFOBT Sampling Set (10 mg/2.0 mL)	QuikRead go	15.0 to 200.0	15.0	15.0	-
SENTiFIT-FOB Gold	Sentinel Diagnostics (Milan, Italy)	SENTiFIT pierceTube (10 mg/1.7 mL)	SENTiFIT 270 analyzer	1.7 to 129.88	17.0	17.68	1.7

Abbreviations: ELISA=Enzyme-linked immunosorbent assay; FIT, Fecal immunochemical test; Hb, Haemoglobin

* For QuikRead go iFOBT it was not possible to set the threshold below 15μg/g to yield 93% specificity; For SENTiFIT-FOB Gold it was only possible to set a specificity of 93.3% at the lowest possible threshold value of 1.7 μg/g.

Table S2 Partial area under the curve [% (95% CI)] for detection of advanced neoplasms by sex and by age

FIT brand	Sex			Age		
	**Female**	**Male**	**p**	**50-64 years**	**65-79 years**	**p**
IDK Hb ELISA	65.4 (59.4-72.2)	63.8 (58.8-69.4)	0.70	66.0 (60.7-72.5)	63.9 (58.6-69.8)	0.60
QuantOn Hem	61.9 (56.4-68.6)	63.5 (58.2-69.0)	0.70	63.8 (58.6-69.6)	63.8 (58.3-70.0)	1.00
immoCARE-C	65.9 (59.8-72.5)	63.8 (58.8-69.7)	0.62	65.9 (60.4-71.7)	64.7 (59.3-71.3)	0.78
CAREprime	64.1 (58.5-70.5)	62.7 (58.2-67.9)	0.72	63,6 (58.5-69.2)	64.1 (58.7-69.9)	0.90
RIDASCREEN Hb	65.8 (59.8-72.5)	63.4 (58.2-69.3)	0.58	66.5 (61.0-72.7)	63.8 (58.4-70.1)	0.54
Eurolyser FOB test	60.9 (55.8-66.4)	62.4 (58.0-67.3)	0.66	62.2 (57.5-67.1)	63.6 (58.8-68.6)	0.70
OC-Sensor	63.5 (57.5-69.8)	62.7 (57.9-67.9)	0.84	63.8 (58.7-68.9)	63.4 (57.9-69.5)	0.94
QuikRead go iFOBT	56.9 (52.9-61.2)	60.0 (56.4-64.0)	0.30	59.4 (55.3-63.5)	59.4 (55.5-63.7)	0.98
SENTiFIT-FOB Gold	60.5 (55.6-65.6)	61.4 (57.2-66.1)	0.79	60.3 (56.1-65.0)	64.1 (59.3-69.0)	0.28

Abbreviations: CI, Confidence interval; FIT, Faecal immunochemical test; Hb, Haemoglobin
